# The Healthy Aging Initiative (HAI): an interdisciplinary longitudinal cohort study to characterize and promote healthspan in senior housing

**DOI:** 10.3389/fpubh.2025.1671875

**Published:** 2025-11-28

**Authors:** Yael Koren, On-Yee Lo, Davide Balos Cappon, Maggie Syme, Heather Andrews, Talia Gilfix, Molly Quigley, John Woolley, Megan Munn, Benjamin Stein, Brad Manor, Thomas G. Travison, Lewis Arnold Lipsitz, Alvaro Pascual-Leone

**Affiliations:** 1 Department of Medicine, Harvard Medical School, Boston, MA, United States; 2 Hinda and Arthur Marcus Institute for Aging Research, Boston, MA, United States; 3 Center for Health Outcomes and Interdisciplinary, Massachusetts General Hospital, Boston, MA, United States; 4 Harvard T. H. Chan School of Public Health, Boston, MA, United States; 5 New York Medical College, Valhalla, NY, United States

**Keywords:** aging, longitudinal cohort, cognition, mobility, healthspan, senior housing, lifestyle, social

## Abstract

**Objective:**

We present the rationale, design, and methods for the Healthy Aging Initiative (HAI), a prospective longitudinal cohort study of older adults primarily residing in senior housing, aimed to identify and characterize factors associated with a prolonged healthspan in this population.

**Methods:**

The HAI, developed with input from experts at the Marcus Institute for Aging Research and informed by a pilot study, includes a community engaged recruitment method and a yearly assessment encompassing the following domains: sociodemographic information, medical history, lifestyle, psychological well-being, physical and cognitive health, mobility, and sensory health. Recruitment is ongoing and includes participants who are aged ≥55 years recruited from six senior housing communities. A control group of community-dwelling participants living in conventional housing aged ≥55 is also planned.

**Results:**

Recruitment remains ongoing. Expected results include characterizing the sociodemographic and health profiles of older adults in independent-living (IL) senior housing, identifying psychological, lifestyle and biological factors associated with a prolonged healthspan, and defining high-risk subgroups to inform future targeted interventions and health promotion strategies. Data will be made publicly available.

**Discussion:**

Longitudinal studies on aging often face challenges such as retention and sustained community engagement. Through a collaborative effort among research scientists in the aging field, housing providers, and older adults, this project aims to collect data on aging trajectories among older adults residing in IL senior housing to inform future initiatives that promote prolonged healthspan in this population.

## 1 Introduction

Populations worldwide are aging rapidly, with over one billion people currently aged 60 and older, leading to significant societal implications (1, 2). Extending healthspan (years spent in good health) by preserving physical, mental, and social functioning as close as possible to end of life is crucial to improving quality of life of older adults and reducing healthcare costs (3, 4).

A potential avenue to support healthspan is through senior housing communities. As of 2024, at least 2.1 million older adults reside in senior housing in the United States (5, 6). Compared to those living independently in single-family homes, older adults in senior housing communities typically receive more support and care (e.g., meal plans, transportation, medical care), enjoy more social interaction (e.g., built-in social activities, communal spaces), and benefit from greater accessibility (e.g., elevators, ramps, grab bars). Residents access to meals, social engagement, transportation, and health related supports within their housing site may reduce ambulance transfers and emergency department (ED) visits (7). Individuals who choose supportive housing settings - though still independent - often already exhibit deterioration in health. As a result, their healthspan trajectory should be conceptualized differently: not simply as living without disease, but as maintaining functional independence (i.e., remaining free from limiting disability) while avoiding substantial acute events associated with chronic disease burden. However, few studies have focused on characterizing healthspan-promoting or modifiable factors (internal and external) associated with prolonged healthspan trajectories among diverse older adults living in this housing arrangement, particularly using longitudinal observational cohorts. Current studies on senior housing and health outcomes also tend to have small sample sizes and limited diversity (8). A recent scoping review highlighted this disparity (8). Furthermore, comprehensive information on residence of affordable or subsidized senior housing remains scarce, highlighting the need for additional longitudinal research in this population (8).

**Figure 1:**
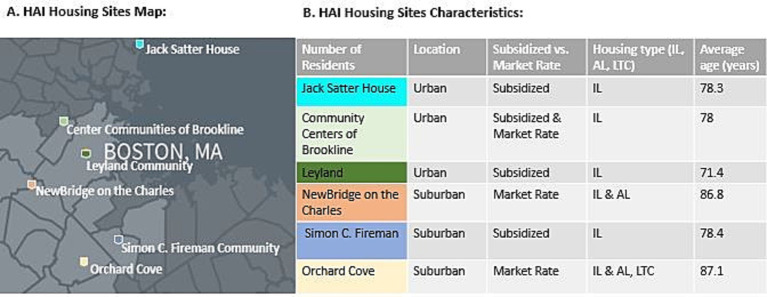
Overview of HAI study housing sites and characteristics. **(A)** Map of six housing site locations across the Greater Boston area: Jack Satter House (Satter) in Revere, Community Centers of Brookline (CCB) in Brookline, Leyland Community (Leyland) in Dorchester, NewBridge on the Charles (NBOC) in Dedham, Orchard Cove (OC) in Canton, and Simon C. Fireman Community (Fireman) in Randolph. **(B)** IL, independent living; AL, assisted living; LTC, long term care.

Hebrew SeniorLife (HSL), founded in 1903 with a tradition of innovation in housing and care for older adults, is uniquely positioned to address these complex issues through the launch of the Healthy Aging Initiative (HAI), an interdisciplinary, community-engaged, longitudinal cohort study. This HAI study unites scientists, residents, and staff from senior housing communities within the same institution and aims to gain deeper insights into the health trajectories of older adults living in senior housing. HSL offers a comprehensive range of services in healthcare, housing, research, and education. The study operates in six senior housing sites which cover a diverse range of functional and financial needs, with both market-rate and subsidized housing options available in urban and suburban settings (Figure 1). HSL also supports community-dwelling older adults with services like home therapy, visiting nurse care, and memory health clinic. In addition, HSL is home to the internationally recognized Hinda and Arthur Marcus Institute for Aging Research, where scientists from multi-disciplinary backgrounds focus on translating research into clinical practice.

The HAI aims to examine how modifiable risk factors shape aging trajectories of senior housing residents and to translate these findings into clinical and community practice. The specific goals of the HAI are to: (i) identify key factors and individual predictors to life-long health and well-being, (ii) determine factors influencing disease onset and progression that may lead to impairments and disability, (iii) discover risk indicators for specific illnesses among older adults living in senior housing facilities. The project also leverages the unique HSL communities to address questions related to housing characteristics —such as subsidy status (subsidized vs. market-rate) and geographic setting (urban vs. non-urban)—, and to compare aging trajectories of senior housing residents with those living independently in the broader community.

Prior to rolling out the HAI protocol across all six HSL housing sites, we piloted the study at two sites – one market-valued and one subsidized. Based on feedback from the residents and staff, we revised the protocol and officially launched the project across all HSL housing communities. We plan to make the data publicly available to researchers to support and advance scientific inquiry in this field.

This paper presents the comprehensive HAI methods and protocol - updated based on feedback from pilot efforts – currently being implemented across the HSL communities. It also discusses strategies for community engagement and potential recruitment challenges in longitudinal studies.

## 2 Methods

**Figure 2:**
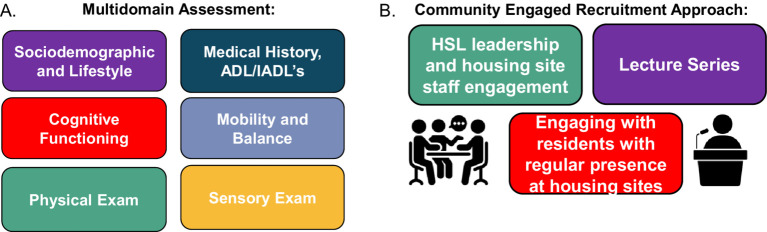
Overview of HAI study domains and community engaged recruitment approach. **(A)** The HAI encompasses six assessment domains. Details for each domain are described in Table 2. **(B)** Three main community engaged recruitment strategies utilized.

The HAI longitudinal cohort study, launched in March 2023, consists of a multi-domain assessment and community engaged retention and recruitment strategies across all HSL housing sites (Figure 2). Senior housing residents within the HAI study are recruited from the following six geographically different housing sites: three urban, and three suburban in the greater Boston area. Additionally, three of the housing sites are primarily subsidized housing while two are market rate, and one is mixed (market and subsidized housing residents) (Figure 1). The average age of older adults living HSL housing is 87 years. Additionally, the resident population at HSL housing sites is expanding in diversity, with two sites now having over 45% Black residents and two others with more than 5% Hispanic residents.

All study materials and procedures were approved by the HSL Institutional Review Board (IRB). Participant data is housed within REDCap, a secure platform for storing data and generating reports (9).

Our recruitment plan envisions recruiting 300 independent housing residents in the first two years, whilst setting up an IRB-approved process such that any new resident moving into independent housing at an HSL community will be entered into the HAI with the option to opt-out. In addition, we plan to recruit an equal number of participants from the community. Overall, over 5 years we aim to recruit 80% of independent housing residents across all HSL Living Communities, targeting approximately 1,000 participants, along with an equal number of community dwelling older adults. With 800 independent housing residents and 800 community-dwelling older adults, we expect to meaningfully characterize participants across functional and health levels comparing housing-site residents and community older adults. This effort also allows us to begin characterizing participants across different key demographic subgroups.

### 2.1 Inclusion and exclusion criteria

Participants are either (1) residents aged 55 and older from one of the six living communities within HSL, or (2) residents aged 55 and older living in separate single-family homes, representing the non-HSL community and serving as the control group (Table 1). This control group will comprise community-dwelling individuals from the Greater Boston area with the potential to understand differences in aging trajectories among senior housing or separate community dwelling older adults. We plan to recruit community-dwelling older adults through open community events. Through an anticipated large cohort, we aim to retrospectively match community-dwelling participants to senior housing residents based on age, sex, education, socioeconomic status, and health characteristics, while accounting for factors that may influence housing decisions such as family or partner support, functional status, and neighborhood environment. These variables will be considered during both the matching process and the interpretation of outcomes when comparing the two populations. We selected an age threshold of 55 and older because residents at HSL housing sites are eligible to move in at that age. Including participants aged 55 and older also allows for a longer observation period. Despite this lower inclusion threshold, most of our residents are over 65. For analytical purposes, we have the option to restrict the sample to a certain age range, when needed, to facilitate comparisons with other larger cohorts.

**Table 1:** HAI inclusion and exclusion criteria.

Inclusion criteria
Individuals aged ≥55 residing at one of the six HSL housing communities; or Non-residents of senior housing sites, individuals aged ≥55 from the Greater Boston area
Exclusion criteria
Legal blindness Deaf or severely hard of hearing Inability to speak English

Exclusion criteria for participating include legal blindness, deafness, and inability to speak and understand English, as these conditions limit the ability to complete the comprehensive in-person assessment (Table 1). To increase inclusivity, we revised the pilot study’s criteria by removing wheelchair use as an exclusion and adapting the assessment to better accommodate individuals with mobility limitations. Although non-English speakers are currently excluded, we plan to translate HAI materials into additional languages to enhance inclusivity and diversity. We have now secured funding to translate the materials into Russian and have hired a Russian-speaking staff member to support this effort. Building on this model, we intend to subsequently expand the project to other languages, such as Spanish and Chinese.

### 2.2 HAI assessment measures

The HAI assessment consists of a self-reported survey and an in-person assessment battery (Table 2). Based on the feedback collected during the pilot project, we shortened the in-person assessment to one hour and selected the most relevant components through discussions among scientists at the Marcus Institute for Aging Research. The following sections describe the content collected through the survey and the in-person assessment battery.

**Table 2:** HAI year 1 in-person assessment domains and tools.

Domain	Assessment tool	Description
Survey		
Socio-demographic information		Age, gender assigned at birth, race/ethnicity, education, occupation (previous/current), primary/additional language, years residing in senior housing
Past medical history	Any medical history of diseases or chronic health conditions as diagnosed by a healthcare professional	Cardiac conditions, hypertension, high cholesterol, diabetes, mental illness, osteoarthritis, rheumatoid arthritis, gout chronic kidney disease, liver disease, cancer
Activities of daily living (ADLs)/Instrumental activities of daily living (IADLs) and lower extremity physical function	Katz Index of ADL; Lawton Instrumental activities of daily living (IADL); Nagi and Rosow-Breslau activities (physical function)	Assessment of functional limitation of daily activities
Lifestyle	Lifestyle questionnaire	Modifiable lifestyle factors: sleep, exercise, diet, social and cognitive engagement, sense of purpose
Advanced care planning		Health care proxy, living will
Fall risk	Stay independent questionnaire	Falls in the past year, recent eye exam, medication use (for sleep or mood)
Mental health	PHQ-4	Anxiety/depression
Senior housing site services	Frequency of utilization questionnaire	Frequency of senior housing social and recreational activities, wellness programs, on-site health services, dining services, spiritual services, and housekeeping, maintenance, and laundry services.
In person assessment battery		
Cognitive	Mini-Cog	Short-term memory/executive function
	Trail making test (Part B)	Executive function
	Verbal fluency	Category verbal fluency (animals)
	RAVLT	Short-term/delayed memory
	Orientation	Orientation items from MoCA
Physical exam	Height/weight	
	Blood pressure and pulse	Seated and Standing/orthostatic hypotension assessment
	Max handgrip	Measured via a dynamometer
Mobility and balance	Dual-task gait	Normal and Dual-Task Gait Assessment
Sensory exam	Visual acuity	Rosenbaum Pocket Vision Screener
	Contrast sensitivity	Pelli-Robson contrast sensitivity chart
	Hearing	Screening conducted with portable audiometer

#### 2.2.1 HAI surveys

Surveys are administered to participants either in person, over the phone with a research assistant, or via email for completion on their own computer at home (Table 2). The survey gathers information on sociodemographic information, including age, gender, sex, race/ethnicity, education, occupation, marital status, number of years residing in senior housing, as well as medical history, including medical and chronic conditions confirmed by a medical provider. To evaluate anxiety and depression, participants complete the Patient Health Questionnaire for Depression and Anxiety (PHQ-4), a brief, validated four-item tool (10). Next, participants are asked about their abilities in activities of daily living (ADL), which includes essential skills for meeting basic physical needs, such as bathing, toileting, dressing, eating, moving, transferring (11). They are also asked about instrumental activities of daily living (IADLs), which encompass skills required to live independently, such as meals preparation, financial management, housekeeping, and shopping (12) and lower extremity mobility in respect to walking and stair climbing (13). The survey features a lifestyle questionnaire, which is a shortened 35-item assessment designed to evaluate modifiable risk factors in aging research (14, 15). This lifestyle questionnaire was developed as part of the Barcelona Brain Health Initiative, a longitudinal cohort study assessing predictors of brain health (14, 15). It evaluates lifestyle domains such as cognitive stimulation and reserve, memory concerns, alcohol and tobacco use, familial history of longevity, hearing loss, medication use, diet, physical activity, loneliness, sleep, social engagement, sense of purpose, and life satisfaction (14, 15). In addition, the survey includes an abbreviated version of the validated Stay Independent Questionnaire to assess fall risk (16) and questions about advance care planning. The advanced care planning section asks whether participants have (1) designated a health care proxy and (2) completed a formal written document specifying desired treatments if they were to become seriously ill.

Participants are asked questions on housing site service utilization to assess the extent to which senior housing residents engage with various service domains. These domains include social and recreational activities, wellness programs (e.g., fitness and health promotion), on-site health services (e.g., primary care, physical therapy, occupational therapy, nursing assistance), dining services, spiritual services, and housekeeping, maintenance, and laundry services. Frequency of use is measured on an ordinal scale: never, once in the past year, 2–3 times in the past year, every few months, monthly, weekly, and daily. An additional response option, “not offered at my housing site,” is provided to account for services unavailable to some residents. Residents are also asked whether they have a point staff person at their housing site which they discuss about health and wellness topics, a relationship established by the Right Care, Right Place, Right Time program and resident services (7).

#### 2.2.2 HAI in-person assessment battery

Similarly, based on participant feedback from the pilot study and discussions among scientists at the Marcus Institute for Aging Research, the following validated, well-established assessments are included in the in-person assessment battery which covers multiple domains, including cognitive, physical and mobility evaluation, sensory examination, and Frailty Index calculation (Table 2). The details for each category are provided below.

##### 2.2.2.1 Cognitive assessment

The cognitive assessments chosen for the annual in-person visit are: (1) the *Mini-Cog*, which assesses short-term verbal memory and executive function via a clock drawing exercise (17); (2) *Semantic Verbal Fluency*, where participants names as many animals as possible within one minute (18); (3) the *Trail Making Test (TMT Part B)*, which assesses cognitive flexibility, task shifting, attention, and visual-motor skills (19); (4) the *Rey Auditory Verbal Learning Test (RAVLT)*, which assesses verbal learning memory (20). During the RAVLT, participants hear a list of 15 nouns and are asked to recall as many words as possible from the list (list a) as possible after five repetitions. This is followed by a second interference list (list b) (20). Participants then immediately recall words from the initial list a, and again after a 20-min delay to measure delayed recall (20). Lastly, (5) we use the orientation items from the *Montreal Cognitive Assessment (MoCA)*, which assesses orientation to date, day of the week, month, year, and place (21).

##### 2.2.2.2 Physical and mobility evaluation

Height and weight are measured in person using standardized equipment to calculate body mass index (BMI). Participants are also asked whether they have experienced any unintentional weight loss of 10 pounds or more in the past year. Blood pressure and pulse are recorded both while seated and one minute after standing to assess for orthostatic hypotension (22). Additionally, maximum handgrip strength is measured using a dynamometer, with two trials for each hand (23).

Gait is assessed under both normal and dual-task conditions using the Mobility Lab device (APDM, Portland, OR). Participants complete two, one-minute walking trials: (1) a normal walking condition, where they walk quietly at their self-selected speed, and (2) a dual-task walking condition, where they walk and concurrently count backward by ones or threes, depending on their ability (24–26). Various gait metrics such as gait speed, stride time variability can be extracted from the Mobility Lab software.

##### 2.2.2.3 Sensory examination

The two visual exams conducted during the in-person assessment are: (1) *the Rosenbaum Pocket Vision Screener,* which is widely used to determine visual acuity in clinical practice (10) and (2) the *Pelli-Robson Contrast Sensitivity Chart*, which is used to measure contrast sensitivity, focusing on the capacity to discern low-contrast objects (27).

For the hearing assessment, we identify diagnosed hearing loss through self-reported hearing aid prescriptions and perform a brief screening with a portable audiometer for participants who do not use hearing aids.

##### 2.2.2.4 Frailty index (FI) calculation

The measures collected from the survey and in-person assessment enable us to calculate the Frailty Index (FI), a tool that evaluates an individual’s frailty level based on their medical history, functional status, and nutritional health (28–30). The Frailty Index ranges from 0 to 1, with higher scores indicating a greater degree of frailty. The following categories are recognized in clinical practice and research: robust (less than 0.15), pre-frailty (0.15 to less than 0.25), mild frailty (0.25 to less than 0.35), moderate frailty (0.35 to less than 0.45), severe frailty (0.45 to less than 0.55), and advanced frailty (0.55 or higher) (29, 30). The Frailty index will serve as both a primary outcome for baseline and follow-up assessments, and as a covariate in analyses of other clinical outcomes to adjust for baseline frailty. Although the primary data collection protocol is intended to minimize missing data – more effectively than reliance on electronic medical records - some missing data may still occur. When this happens, we will use multiple imputation methods as appropriate to handle missing data.

### 2.3 HAI report

Recent longitudinal cohort studies, such as All of US, have utilized return of results to foster trust with participants, and as a recruitment strategy (31). This is a result of research participants expressing a desire to receive personal health information. As part of our recruitment and reciprocity efforts, we developed the HAI Participant Research Report. This report is designed to help study participants better understand their assessment results and gain insights into health behaviors that could support their overall well-being.

The report offers research results for each domain, comparing them to normal ranges based on sex and age and includes explanations of each domain and its significance to healthy aging. To develop this report, we collaborated with HSL geriatricians, primary care physicians, and participants from the pilot study. Based on their collective feedback, we revised the report to incorporate empowering and reassuring language, aiming to enhance its impact and clarity for participants. The HAI report is personalized for each individual based on data collected during their assessment and emphasizes that the results are intended for the context of research rather than a clinical evaluation. The report also aims to promote conversation with the participants’ primary care providers.

Sections of the HAI report include the domains described in the assessment: (i) physical health, (ii) cognitive health, (iii) gait and mobility, (iv) hearing, (v) vision, (vi) mental health (anxiety and depression), (vii) lifestyle habits affecting brain health, and (viii) a summary (Supplementary Appendix A includes the cognitive health example). To manage the boundary between research feedback and clinical care, we will ensure that participants understand that research reports do not constitute a clinical diagnosis. Participants will be advised to consult appropriate healthcare providers if significant concerns are identified. Our research team also works closely with staff at residential housing sites, who can support residents in accessing the services they need. In particular, within our organization, several outpatient services – such as the Wolk Center for Memory Health – are available for participants to receive further assessment and support.

To assess how participants engaged with study recommendations, we track whether they opted to receive research reports. During annual follow-up assessments, we document any self-reported behavior changes attributed to the reports, as well as whether participants discussed the content with their primary care provider or a specialist. This information will serve as a measure of participant engagement and inform future longitudinal analyses.

### 2.4 Community engaged recruitment

We implemented the following three strategies to recruit residents from the HSL housing sites: partnering with HSL housing community staff, an HAI lecture series and blog, and initiating an HAI participant ambassador group.

#### 2.4.1 HAI partner housing communities staff

We collaborate closely with HSL staff such as housing directors, resident care coordinators and nurses from the six housing communities to strengthen connections and adapt our recruitment efforts to meet the specific culture of each housing site. We also developed a quarterly newsletter to update the directors of each site on the latest developments and progress of the HAI.

To obtain direct feedback from participants and build mutual trust, we recruited **s**tudy ambassadors as volunteers to act as liaisons between the initiative’s research team and their community. Their role involves community engagement and outreach, facilitating direct communication between the HAI team and the residents. The ambassadors also provide valuable feedback on the HAI’s recruitment strategies and represent community perspectives and preferences, ensuring that the initiative remains responsive and adaptable to the needs of its participants. This feedback loop helps refine recruitment approaches and enhance participant engagement, trust and satisfaction (32).

#### 2.4.2 HAI lecture series and online blog

We established the HAI lecture series based on HAI ambassador feedback. The HAI lecture series serves as community engaged recruitment and HAI study participant engagement. Led by Marcus faculty scientists and experts in aging, these lectures are conducted at housing sites and focus on the latest evidence-based research and scientific discoveries that support healthy aging. Key topics covered include brain health screenings, mitigating cardiovascular risk, optimizing nutrition, emotional well-being, strategies for fall risk reduction, and mental health awareness.

We also developed a HAI study website which houses the lecture series recordings, and a healthy aging blog, to make these educational resources more accessible to the broader community.

## 3 Anticipated results

We anticipate that the HAI will yield several key findings. First, we expect to generate a detailed characterization of the sociodemographic and health profiles of older adults residing in senior congregate housing. These findings will allow for comparisons across different housing site characteristics, including market-rate versus subsidized housing and urban versus suburban settings. We will conduct multi-level modeling to account for potential clustering effects arising from the variation in housing site types. Second, through longitudinal analyses, we aim to identify factors associated with prolonged healthspan, providing insights into trajectories of healthy aging within this population which may inform future interventions within housing sites. Finally, we expect to identify subgroups at elevated risk for adverse health outcomes who may benefit from targeted preventive or therapeutic interventions. We intend these results to inform future research and guide the development of tailored health promotion strategies for older adults in senior housing communities. Additionally, through data sharing, we aim to provide a valuable resource that can support further research in this field.

## 4 Discussion

The Healthy Aging Initiative (HAI) is a longitudinal cohort study that integrates community engaged recruitment and interdisciplinary collaboration to better characterize and understand factors associated with prolonged healthspan among older adults residing in senior housing. As the number of older adults continues to grow and more individuals utilize senior housing, it is important to invest in understanding their health trajectories to better support their needs and enhance senior housing models (8). One cohort study conducted in Finland called the BoAktiv senior house survey, evaluated elements of active aging in senior housing residential cohort (33). The HAI’s focus on understanding health trajectories and factors associated with prolonged healthspan in senior housing is unique for longitudinal cohort studies in the United States. For example, the National Health and Aging Trends Study (NHATS) predominantly focuses on older adults living in community settings, with only about 7.1% of NHATS participants in 2021 residing in senior housing (34). Importantly, a large body of research conducted by the National Opinion Research Center (NORC) has valuably examined health outcomes of senior housing residents compared to older adults living in the community over a two-year period (35). The HAI is distinguished by its focus on senior housing residents and individual markers of healthy aging, while incorporating community engagement, ambassador groups and the return of research results. For example, our active presence at each housing site, together with the integration of the Marcus institute in HSL, helps bridge gaps between the research center and residents. Additionally, we collaborate with housing site staff, who maintain strong connections with residents - may allow us to capture health trajectories of more socially isolated older adults who might not initially seek research participation.

Of note, loss to follow-up is more common among longitudinal studies of older adults due to health challenges such as illness, unexpected hospitalizations, and challenges with remote communication, and engagement (36). To address these challenges, greater investment in strategies to enhance participant engagement is essential. To recruit and engage participants, we collaborate closely with housing staff to understand unique housing site residents’ preferences for engagement. As a result of previous focus groups, and pilot study planning, we engage housing residents via a lecture series and study ambassadors. The establishment of ambassadors and the development of the HAI lecture series represent novel and innovative strategies for fostering an informed and engaged participant community, which may build trust with participants, and help participants remain actively engaged throughout the study (32, 36). These efforts are designed to address the challenges of participant engagement and retention and ensure that the cohort remains robust across study phases. We plan to study this effort over time and adjust recruitment strategies as necessary. In particular, we will collect information to calculate retention rates and identify barriers to follow-up, allowing for a more accurate evaluation of the impact of our engagement strategies. This will include reasons for dropping out - such as health deterioration, hospitalization, loss of interest, busy schedule, relocation, or other factors- to better distinguish among contributing factors. For participants who are not reachable, we will communicate with housing site staff to ascertain potential reasons.

### 4.1 Future directions

Our established relationship with the HSL community and growing HAI cohort has the potential to share data with other scientists to advance aging research and expand data collection to topical observational sub-studies, along with the incorporation of bio specimen collection (37). Additionally, as we expand our cohort to older adults residing in the community setting, we hope to examine differences in factors associated with prolong healthspan between older adults in senior housing and those residing independently in the neighboring community. A recent study highlighted that community dwelling US older adults with caregivers at home, were more likely to experience an unmet care need event, compared to older adults residing in residential care potentially due to inconsistent caregiver availability, varying levels of caregiver training in the community setting (38). In contrast, residential care settings often provide structured and continuous support, potentially reducing the likelihood of unmet care needs. However, to realize the potential of senior housing to improve access to health promotion and aging resources, further research is needed to predict the relevant modifiable factors associated with a longer healthspan for specific individuals. Notably, senior housing care originally developed to provide functional support and manage chronic diseases. However, contemporary senior housing communities are evolving beyond this traditional model, leveraging environmental factors and offering an array of guided and supported activities to actively promote health and prevent disease. The HAI study has the potential to identify high-risk populations within supportive housing environments, informing more precise and targeted interventions to improve health outcomes and maintain independence. Furthermore, by examining the influence of resident engagement and housing site characteristics, our findings may inform interventions to promote health equity within senior housing and beyond.

### 4.2 Limitations

The HAI study includes a novel, underrepresented population of older adults living in senior housing in diverse settings. Some limitations include that HSL housing is a unique institution which provides supportive services within senior housing, limiting generalization to similar senior housing models. We also aim to recruit a control group of age, sex and health condition-matched participants. There is a potential for unmeasured differences between senior housing residents and community-dwelling participants residing in separate, single-family homes that could influence aging outcomes, such as underlying social support networks or personal preferences in housing decisions, which may not be fully accounted for even after matching. Additionally, the inclusion of a research report containing health promotion tips may influence participant outcomes, potentially biasing the observation of natural health trajectories. To account for this, we will use follow-up data collected on reported behavior changes and medical services outreach related to the report as covariates in our longitudinal analyses.

## 5 Conclusion

The HAI integrates multi-domain gold-standard assessments, interdisciplinary scientific input, and a community engaged recruitment approach in senior housing. To recruit participants the HAI uniquely provides a return of research results to participants. Future recruitment efforts will focus on strengthening community engaged strategies and partnership with housing staff. We anticipate that longitudinal data collection and analysis of temporal patterns will fortify the initiative’s capacity to discern health trajectories, particularly by uncovering factors that influence healthy aging among senior housing residents.

## Data Availability

The original contributions presented in the study are included in the article/supplementary material, further inquiries can be directed to the corresponding author/s.
