# Supplementary material for: The Healthy Aging Initiative (HAI): an interdisciplinary longitudinal cohort study to characterize and promote healthspan in senior housing
Source: Front Public Health. 2025 Nov 28;13:1671875. doi: 10.3389/fpubh.2025.1671875 (PMC12699381; doi:10.3389/fpubh.2025.1671875)
Supplement: Supplementary file 1 [file Data_Sheet_1.docx]

**Appendix A**

**HAI Participant Report Template (Sample Section)**

**Cognitive HEALTH**

Cognitive SCREENING

You completed a screening of your cognitive health, including tests of overall cognitive function, memory, executive function and visuospatial skill. A cognitive screening can help identify areas for potential follow-up, but is not a comprehensive test of these cognitive domains. When comparing your scores to those within your age group and level of education, your screening indicates you are functioning **[within, somewhat below, significantly below] the expected range.**

| **Domain** | **Functional Category** |
| --- | --- |
| Overall Cognition | [insert level here] |
| Memory | [insert level here] |
| Executive Function | [insert level here] |

**[Within the expected range]** We recommend that you re-check your cognitive function annually, and if you notice any changes or have concerns that come up along the way you should discuss this with your primary care provider.

**[Somewhat below the expected range]** [Single test or domain below -1.5, mild impairment] We recommend that you review these screening results with your primary care provider in case these results with your overall medical history indicate a need for more comprehensive cognitive testing. If so, they will be able to refer you to the appropriate specialist, such as a neuropsychologist.

**[Significantly below the expected range]** [Multiple tests/domains below -1.5, moderate or severe impairment] We strongly recommend that you review these screening results with your primary care provider. If needed, they will be able to refer you to the appropriate specialist for a more comprehensive cognitive assessment, such as a neuropsychologist or neurologist.

*Resources:*

To learn more about cognition in aging and how you can maintain the health of your brain, visit these additional resources:

<https://www.hebrewseniorlife.org/blog/navigating-mild-cognitive-impairment-prevention-and-treatment>

<https://www.hebrewseniorlife.org/blog/normal-memory-loss-or-alzheimers>

<https://www.health.harvard.edu/mind-and-mood/train-your-brain>
